# OncomiRs: the discovery and progress of microRNAs in cancers

**DOI:** 10.1186/1476-4598-6-60

**Published:** 2007-09-25

**Authors:** William CS Cho

**Affiliations:** 1 Department of Clinical Oncology, Queen Elizabeth Hospital, Room 1305, 13/F, Block R, 30 Gascoigne Road, Kowloon, Hong Kong SAR, PR China

## Abstract

microRNAs (miRNAs) are evolutionarily conserved, endogenous, small, noncoding RNA molecules of about 22 nucleotides in length that function as posttranscriptional gene regulators. They are deemed to play a crucial role in the initiation and progression of human cancer, and those with a role in cancer are designated as oncogenic miRNAs (oncomiRs). For example, *miR-15 *and *miR-16 *induce apoptosis by targeting *Bcl2*. miRNAs from the *miR-17-92 *cluster modulate tumor formation and function as oncogenes by influencing the translation of E2F1 mRNA. *miR-21 *modulates gemcitabine-induced apoptosis by phosphatase and tensin homolog deleted on chromosome 10-dependent activation of PI 3-kinase signaling. *miR-34a *acts as a suppressor of neuroblastoma tumorigenesis by targeting the mRNA encoding E2F3 and reducing E2F3 protein levels. The chromosomal translocations associating with human tumors disrupt the repression of High mobility group A2 by *let-7 *miRNA. In addition, the oncomiRs expression profiling of human malignancies has also identified a number of diagnostic and prognostic cancer signatures. This article introduces the roles of oncomiRs in neoplasm development, progression, diagnosis, prognostication, as well as their mechanism of actions on target mRNAs and the functional outcomes of their actions on mRNAs. The paper ends with a brief perspective to the future of oncomiRs.

## Introduction

microRNAs (miRNAs) are evolutionarily conserved, endogenous, small, noncoding RNA molecules of about 22 nucleotides in length that function as posttranscriptional gene regulators [1]. They are encoded in the genome and are generally transcribed by RNA polymerase II. miRNAs work via RNA-induced silencing complexes, targeting them to messenger RNAs where they either repress translation or direct destructive cleavage [2]. Recent evidences have shown that miRNAs have diverse functions, including the regulation of cellular development, differentiation, proliferation and apoptosis. The first miRNA was described in 1993, in which the *C. elegans *heterochronic gene *lin-4 *encoded small RNAs with antisense complementarity to *lin-14 *[3]. It is estimated that vertebrate genomes encode up to 1,000 unique miRNAs [4], which are predicted to regulate expression of at least 30% of genes [5]. Though more than 530 miRNAs have been identified in human, much remains to be understood about their precise cellular function and role in the development of diseases. Recent evidences indicate that miRNAs can function as tumor suppressors and oncogenes, and the miRNAs with a role in cancer are designated as oncogenic miRNAs (oncomiRs). This article introduces the roles of oncomiRs in neoplasm development, progression, diagnosis, prognostication, as well as their mechanism of actions on target mRNAs and the functional outcomes of their actions on mRNAs.

## microRNAs experiment

miRNA microarray is a high-throughput approach to study the expression of miRNAs in cultured cells or tissues. Unlike the traditional cDNA microarray expression profiling, RNA samples used for miRNA microarray hybridization need to be enriched for small RNAs. Usually, the first step of a miRNA microarray experiment is the isolation of total RNA and the enrichment or direct isolation of small RNA. The miRNAs are then labeled and cleaned-up, following with miRNA hybridization to arrays spotted with miRNA probes. After washes and scanning, the differential miRNAs are identified. Subsequent validation is recommended using Northern blot, quantitative reverse transcription-polymerase chain reaction (qRT-PCR), or other analytical methods. The recent development of highly efficient labeling method and novel microarray probe design enable direct label as low as 120 ng of total RNA using Cy3 or Cy5, without fractionation or amplification, to produce precise and accurate measurements that span a linear dynamic range from 0.2 amol to 2 fmol of input miRNA. The assay is also applicable for formalin-fixed paraffin-embedded samples [6].

## microRNAs and cancers

It has been reported that miRNAs play a crucial role in the initiation and progression of human cancer. Deregulation of oncomiRs is associated with genetic or epigenetic alterations, including deletion, amplification, point mutation and aberrant DNA methylation [7]. Their expression profiling of human malignancies has identified signatures involving in cancer development, progression, diagnosis and prognosis (Table 1).

**Table 1:** The role of microRNAs in cancers

**microRNAs**	**Tumorigenesis**	**Diagnosis**	**Prognosis**
*miR-9*	Neuroblastoma		
*miR-10b*	Breast cancer		
*miR-15, miR-15a*	Leukemia, pituitary adenoma		
*miR-16, miR-16-1*	Leukemia, pituitary adenoma		
*miR-17-5p, miR-17-92*	Lung cancer, lymphoma		
*miR-20a*	Lymphoma, lung cancer		
*miR-21*	Breast cancer, cholangiocarcinoma, head & neck cancer, leukemia, cervical cancer		Pancreatic cancer
*miR-29, miR-29b*	Leukemia, cholangiocarcinoma		
*miR-31*	Colorectal cancer		
*miR-34a*	Pancreatic cancer		Neuroblastoma
*miR-96*	Colorectal cancer		
*miR-98*	Head & neck cancer		
*miR-103*	Pancreatic cancer		
*miR-107*	Leukemia, pancreatic cancer		
*miR-125a, miR-125b*	Neuroblastoma, breast cancer		
*miR-128*	Glioblastoma		
*miR-133b*	Colorectal cancer		
*miR-135b*	Colorectal cancer		
*miR-143*	Colon cancer, cervical cancer		
*miR-145*	Breast cancer, colorectal cancer		
*miR-146*	Thyroid carcinoma		
*miR-155*	Breast cancer, leukemia, pancreatic cancer		Lung cancer
*miR-181, miR-181a, miR-181b, miR-181c*	Leukemia, glioblastoma, thyroid carcinoma		
*miR-183*	Colorectal cancer		
*miR-184*	Neuroblastoma		
*miR-196a-2*			Pancreatic cancer
*miR-221*	Glioblastoma, thyroid carcinoma	Pancreatic cancer	
*miR-222*	Thyroid carcinoma		
*miR-223*	Leukemia		
*miR-301*		Pancreatic cancer	
*miR-376*		Pancreatic cancer	
*let-7, let-7a, let-7a-1, hsa-let-7a-2, let-7a-3*	Lung cancer, colon cancer		Lung cancer

### The role of microRNAs in tumorigenesis

Calin *et al*. first made the connection between microRNAs and cancer by showing that *miR-15 *and *miR-16 *are located at chromosome 13q14, a region deleted in more than half of B-cell chronic lymphocytic leukemia (CLL). Detailed deletion and expression analysis showed that *miR-15 *and *miR-16 *are located within a 30 kb region of loss in CLL, and that both genes were deleted or downregulated in approximately 68% of CLL cases [8]. They further showed that miR genes were frequently located in cancer-associated genomic regions or in fragile sites. The full complement of miRNAs in a genome might be extensively involved in cancers [9]. Bottoni *et al*. found that *miR-15a *and *miR-16-1 *were expressed at lower levels in pituitary adenomas as compared to normal pituitary tissue. Their expression was inversely correlated with tumor diameter and with arginyl-tRNA synthetase expression, but was directly correlated with *p43 *secretion, suggesting that these miRNAs might influence tumor growth [10]. Cimmino *et al*. then demonstrated that *miR-15a *and *miR-16-1 *expressions were inversely correlated to *Bcl2 *expression in CLL and that both miRNAs negatively regulated *Bcl2 *at a posttranscriptional level. *Bcl2 *repression by these miRNAs induced apoptosis in a leukemic cell line model. Therefore *miR-15 *and *miR-16 *were natural antisense *Bcl2 *interactors that could be used for therapy of *Bcl2*-overexpressing tumors [11]. Recently, Garzon *et al*. showed that all-trans retinoic acid (ATRA) downregulation of *Bcl2 *and *Ras *was correlated with the activation of *miR-15a/miR-16-1 *[12].

Amplification and overexpression of the *miR-17-92*, which comprised 7 miRNAs and resided in intron 3 of the *C13orf25 *gene at 13q31.3, has been reported with pointers to functional involvement in the development of lymphoma and lung cancer. He *et al*. compared B-cell lymphoma samples and cell lines to normal tissues, and found that the levels of the primary or mature miRNAs derived from the *miR-17-92 *locus were often substantially increased in cancer cells. Their studies indicated that miRNAs could modulate tumor formation and function as oncogenes, implicating the *miR-17-92 *cluster as a potential human oncomicroRNAs (oncomiRs) [13]. O'Donnell *et al*. demonstrated that c-Myc activated expression of a set of 6 miRNAs on human chromosome 13 that was tied to the development of human lymphoma. It was found that the expression of E2F1 was negatively regulated by *miR-17-5p *and *miR-20a *in HeLa cells. Their findings revealed a mechanism through which the c-Myc protein simultaneously activated E2F1 transcription and limited its translation, allowing a tightly controlled proliferative signal [14]. Woods *et al*. proposed a model whereby *miR-17-92 *promoted cell proliferation by shifting the E2F transcriptional balance away from the pro-apoptotic E2F1 and toward the proliferative E2F3 transcriptional network [15].

On the other hand, Hayashita *et al*. found that *miR-17-92 *was markedly overexpressed in lung cancer, especially with small-cell lung cancer. Their findings suggested that marked overexpression of the *miR-17-92 *cluster with occasional gene amplification might play a role in the development of lung cancer and that the *C13orf25 *gene might well be serving as a vehicle in this regard [16]. Matsubara *et al*. showed that inhibition of *miR-17-5p *and *miR-20a *with antisense oligonucleotides could induce apoptosis selectively in lung cancer cells overexpressing *miR-17-92*, suggesting the possibility of oncomiR addiction to expression of these miRNAs in a subset of lung cancers. Their discoveries contributed towards better understanding of the oncogenic roles of *miR-17-92*, which might ultimately lead to the future translation into clinical applications [17].

Iorio *et al*. showed that miRNAs were aberrantly expressed in human breast cancer compared with normal breast tissue, with the most significantly downregulated miRNAs being *miR-10b*, *miR-125b *and *miR-145*, whereas the most significantly upregulated miRNAs being *miR-21 *and *miR-155*. The miRNA expression was also found to be correlated with specific breast cancer biopathologic features, such as estrogen and progesterone receptor expression, tumor stage, vascular invasion or proliferation index [18]. Si *et al*. found that *miR-21 *was highly overexpressed in breast tumors compared to the matched normal breast tissues. They found that the anti-*miR-21*-mediated cell growth inhibition was associated with increased apoptosis and decreased cell proliferation. Their results suggested that *miR-21 *functioned as an oncogene and modulated tumorigenesis through regulation of genes such as *Bcl2 *and it might serve as a novel therapeutic target [19]. Zhu *et al*. performed two-dimensional differentiation in-gel electrophoresis of tumors treated with anti-*miR-21 *and identified the tumor suppressor tropomyosin 1 as a potential *miR-21 *target. Downregulation of tropomyosin 1 in breast cancer by *miR-21 *might explain why suppression of *miR-21 *could inhibit tumor growth, further supporting the notion that *miR-21 *functions as an oncogene [20]. Meng *et al*. identified *miR-21 *was also highly overexpressed in malignant cholangiocytes. Inhibition of *miR-21 *increased sensitivity to gemcitabine, it modulated gemcitabine-induced apoptosis by phosphatase and tensin homolog deleted on chromosome 10-dependent activation of PI 3-kinase signaling [21]. Tran *et al*. found that *miR-21 *was highly expressed in the head and neck cancer cell lines. Several tumor suppressor genes were identified to be potential targets of miRNAs, including kinesin family member 1B isoform alpha, hypermethylated in cancer 2 and pleomorphic adenoma gene 1 [22].

Costinean *et al*. showed that E(mu)-mmu-*miR-155 *transgenic mice exhibited preleukemic pre-B-cell proliferation evident in spleen and bone marrow, followed by frank B-cell malignancy. Their findings indicated that *miR-155 *could induce polyclonal expansion, favoring the capture of secondary genetic changes for full transformation [23]. Using miRNA cloning and qRT-PCR of mature miRNAs, Fulci *et al*. demonstrated that *miR-21 *and *miR-155 *were dramatically overexpressed in CLL patients [24]. Besides, Roldo *et al*. showed that the expression of *miR-103 *and *miR-107*, associating with a lack of *miR-155 *expression, could discriminate pancreatic tumors from normal pancreas. Their results suggested that the alteration in miRNA expression was related to endocrine and acinar neoplastic transformation [25].

Felli *et al*. demonstrated that treatment of CD34+ progenitors with *miR-221 *and *miR-222 *caused impaired proliferation and accelerated differentiation of erythropoietic cells, coupled with the downmodulation of Kit protein. The decline of *miR-221 *and *miR-222 *during exponential erythropoietic growth unblocked Kit protein production at mRNA level, thus leading to expansion of early erythroblasts [26]. He *et al*. also showed that *miR-146*, *miR-221 *and *miR-222 *distinguished unequivocally between papillary thyroid carcinoma (PTC) and normal thyroid. The upregulation of these miRNAs was strongest associated with a dramatic loss of Kit transcript and its protein. Sequence changes in genes targeted by these miRNAs could contribute to the regulation of Kit involved in PTC pathogenesis [27]. Analyzing the genome-wide miRNA expression profile in human PTCs using microarray, Pallante *et al*. detected a significant increase of *miR-181b*, *miR-221 *and *miR-222 *in the comparison of PTCs with normal thyroid tissue. Further confirmation by Northern blot analysis and qRT-PCR, their results suggested miRNA deregulation as an important event in thyroid cell transformation [28].

The analysis of both glioblastoma tissues and glioblastoma cell lines allowed Ciafre *et al*. to identify a group of miRNAs whose expression was significantly altered in the tumor. *miR-221 *was strongly upregulated, whereas *miR-128*, *miR-181a*, *miR-181b *and *miR-181c *were downregulated in glioblastoma [29]. Pekarsky *et al*. discovered that the expression levels of *miR-29 *and *miR-181 *were inversely correlated with *Tcl1 *expression in CLL. Their results showed that *miR-29 *and *miR-181 *might be candidates for therapeutic agents in CLL overexpressing *Tcl1 *[30]. By *in silico *analysis, Mott *et al*. identified a putative target site in the *Mcl1 *mRNA and found that *miR-29b *was downregulated in malignant cells, consistent with Mcl1 protein upregulation. Enforced *miR-29b *expression reduced Mcl1 protein expression in the KMCH cholangiocarcinoma cells, thus *miR-29 *was an endogenous regulator of Mcl1 protein expression [31].

Examined by RT-PCR, Bandres *et al*. identified 13 significantly deregulated mature miRNAs in colorectal cancer, including *miR-31*, *miR-96*, *miR-133b*, *miR-135b*, *miR-145 *and *miR-183*. In addition, the expression level of *miR-31 *was correlated with the stage of colorectal cancer [32]. Akao *et al*. found that *miR-143 *and *miR-145 *expression levels were extremely reduced in the colon cancer cells. The transfection of each precursor miRNA into the cells demonstrated a significant growth inhibition in human colon cancer DLD-1 and SW480 cells, whereas *Erk5 *was determined to be the target gene of *miR-143 *[33].

Laneve *et al*. showed that *miR-9*, *miR-125a *and *miR-125b *acted in an additive manner by repressing the truncated isoform of the neurotrophin receptor tropomyosin-related kinase C. They found that the downregulation of this isoform was critical for regulating neuroblastoma cell growth [34].*In vitro *functional studies of neuroblastoma cell lines indicated that *miR-184 *played a significant role in apoptosis. Chen and Stallings suggested that neuroblastoma derived *Myc *myelocytomatosis viral related oncogene might mediate a tumorigenic effect through directly or indirectly regulating the expression of miRNAs that were involved with neural cell differentiation and/or apoptosis [35].

Welch *et al*. showed that *miR-34a *on chromosome 1p36.23 was generally expressed at lower levels in unfavorable primary neuroblastoma tumors relative to normal tissue. *miR-34a *directly targeted the mRNA encoding E2F3 and significantly reduced E2F3 protein levels. Their results suggested that *miR-34a *acted as a suppressor of neuroblastoma tumorigenesis [36]. Chang *et al*. showed that *miR-34a *was frequently absent in pancreatic cancer cells. They demonstrated that this miRNA was directly transactivated by *p53*. *miR-34a*-responsive genes were highly enriched for those that regulated cell-cycle progression, apoptosis, DNA repair and angiogenesis. It was likely that an important function of *miR-34a *was the modulation and fine-tuning of the gene expression program initiated by *p53 *[37].

Nervi *et al*. found that the expression level of *miR-223 *was correlated with the differentiation fate of myeloid precursors. The activation of both pathways of transcriptional regulation by the myeloid lineage-specific transcription factor CCAAT/enhancer-binding protein-α (C/EBPa) and posttranscriptional regulation by *miR-223 *appeared essential for granulocytic differentiation and clinical response of acute promyelocytic leukemia blasted to ATRA [38]. Using miRNA microarray platform and qRT-PCR, Garzon *et al*. reported the expression of miRNAs in acute promyelocytic leukemia patients and cell lines during ATRA treatment. They found upregulation of *miR-107 *targeted nuclear factor 1-A, a gene involving *miR-223 *and C/EBPa in a regulatory loop during granulocytic differentiation. Besides, ATRA downregulation of *Ras *and *Bcl2 *was shown to correlate with the activation of *let-7a *miRNA [12].

Johnson *et al*. showed that the *let-7 *miRNA family negatively regulated *let-60/Ras*. Loss of *let-60/Ras *suppressed *let-7 *miRNA complementary sites, restricting reporter gene expression in a *let-7*-dependent manner. *let-7 *miRNA expression was lower in lung tumors than in normal lung tissue, while Ras protein was significantly higher in lung tumors, providing a possible mechanism for *let-7 *miRNA in cancer [39]. Akao *et al*. found that the levels of Ras and c-Myc proteins in *let-7 *miRNA low-expressing DLD-1 human colon cancer cells were lowered after the transfection with *let-7a-1 *precursor miRNA, whereas the levels of both of their mRNAs remained almost unchanged. Their findings suggested the involvement of *let-7 *miRNA in the growth of colon cancer cells [40]. Meng *et al*. demonstrated that *let-7a *miRNA was upregulated and contributed to the survival effects of enforced Interleukin-6 activity in malignant human cholangiocytes. It contributed to the constitutively increased phosphorylation of the signal transducers and activators of transcription-3 factors by a mechanism involving neurofibromatosis 2 [41]. Brueckner *et al*. showed that the human *let-7a-3 *precursor miRNA on chromosome 22q13.31 was associated with a CpG island. They identified *let-7a-3 *precursor miRNA as an epigenetically regulated miRNA gene in lung adenocarcinomas with oncogenic function and suggested that aberrant miRNA gene methylation might contribute to the human cancer epigenome [42].

Mayr *et al*. reported that the chromosomal translocations associating with human tumors disrupted the repression of High mobility group A2 (*Hmga2*) by *let-7 *miRNA. They found that the loss of miRNA-directed repression of an oncogene provided a mechanism for tumorigenesis, and disrupting a single miRNA-target interaction could produce an observable phenotype in mammalian cells [43]. Lee and Dutta also demonstrated that ectopic expression of *let-7 *miRNA reduced *Hmga2 *and cell proliferation in lung cancer cell. Their results suggested that some tumors activated the oncogene through chromosomal translocations that eliminated the oncogene's 3' untranslated region with the *let-7 *miRNA target sites [44]. Hebert *et al*. reported that *Hmga2 *expression in head and neck squamous cell carcinoma cells was regulated in part by *miR-98*. They showed that the transfection of pre-*miR-98 *during normoxia diminished *Hmga2 *and potentiate resistance to doxorubicin and cisplatin. Their findings implicated the role of *miR-98 *as a key element in modulating tumors during hypoxia [45].

Stem cells have the ability to escape cell cycle stop signals, which are similar to cancer cells. On the basis of cell cycle markers and genetic interactions, Harfield *et al*. reported that dicer-1 mutant germline stem cells were delayed in the G1 to S transition, which was dependent on the cyclin-dependent kinase inhibitor dacapo, suggesting that miRNAs were required for stem cells to bypass the normal G1/S checkpoint. The miRNA pathway might be part of a mechanism that made stem cells insensitive to environmental signals that normally stop the cell cycle at the G1/S transition [46].

### The role of microRNAs in cancer diagnosis

Lu *et al*. used a bead-based flow cytometric miRNA expression profiling method to present a systematic expression analysis of miRNAs from human cancer samples, including colon, liver, pancreas and stomach cancers. The miRNA expression profiles were able to successfully classify poorly differentiated tumors. Their findings highlighted the potential of miRNA profiling in cancer diagnosis [47].

Analyzing the entire miRNAome in pituitary adenomas and in normal pituitary samples by microarray and RT-PCR, Bottoni *et al*. showed that miRNA expression could differentiate micro- from macro-adenomas and the treated from non-treated patient samples. Several miRNAs were involved in cell proliferation and apoptosis. Predictive miRNAs could be potentially useful diagnostic markers, improving the classification of pituitary adenomas [48].

With the application of *in situ *RT-PCR, Lee *et al*. showed that the aberrantly expressed *miR-221*, *miR-301 *and *miR-376a *were localized to pancreatic cancer cells but not to stroma or normal acini or ducts. Aberrant miRNA expression offered new clues to pancreatic tumorigenesis and might provide diagnostic biomarkers for pancreatic cancer [49].

### The role of microRNAs in cancer prognosis

Takamizawa *et al*. reported that expression of *let-7 *miRNA was frequently reduced in human lung cancers, and that reduced *let-7 *miRNA expression was significantly associated with shorter postoperative survival. In addition, overexpression of *let-7 *miRNA in A549 lung adenocarcinoma cell line inhibited lung cancer cell growth *in vitro*. Their study reported the potential clinical and biological effects of *let-7 *miRNA alteration [50]. Yanaihara *et al*. identified miRNA expression profiles which could discriminate lung cancers from noncancerous lung tissues as well as molecular signatures that differed in tumor histology. High *hsa-miR-155 *and low *hsa-let-7a-2 *precursor miRNA expression were found to be correlated with poor survival of lung adenocarcinomas. Their results indicated that miRNA expression profiles were not just diagnostic markers, but also prognostic markers of lung cancer [51].

Roldo *et al*. showed that the overexpression of *miR-21 *in pancreatic tumors was strongly associated with both high Ki67 proliferation index and the presence of liver metastasis. Their results suggested that the alteration in miRNA expression was related to progression of malignancy [25]. On the other hand, Bloomston *et al*. compared the expression pattern of miRNAs in pancreatic cancer with those of normal pancreas and chronic pancreatitis using miRNA microarrays. Differentially expressed miRNAs were identified which could differentiate pancreatic cancer from normal pancreas, chronic pancreatitis, or both. High expression of *miR-196a-2 *was found to predict poor survival [52].

## Perspective

Discovery of the role of miRNAs in various pathological processes has opened up possible applications in molecular diagnostics and prognostics, particularly for cancer. Some miRNAs are controlled by epigenetic alterations in cancer cells, including DNA methylation and histone modification. Using chromatin modifying drugs to activate tumor suppressor miRNAs can regulate target oncogenes, and it may lead to novel cancer therapies in the future. miRNAs can  complement other genomic and proteomic biomarkers for cancer diagnosis and prognosis [53,54]. Although each miRNA can control hundreds of target genes, it remains a great challenge to identify the accurate miRNA targets for cancer research. On the other hand, as stem cells can regenerate and develop into many different cell types in the body, they are always a focus of intense research interest. It has been reported that miRNA pathway plays a regulatory role in stem cell division, whether this mechanism will contribute to prevent and treat cancer will be worth studying [55].

The Nobel Prize in Physiology or Medicine 2006 was awarded to Andrew Fire and Craig Mello for their discovery of RNA interference (RNAi) – gene silencing by double-stranded RNA. Since regulatory trans-acting antisense RNAs has been found to exist in several species, posttranscriptional regulation of genes is no longer regarded as an odd regulatory mechanism. miRNAs repress their target mRNAs by complementary base pairing and induction of the RNAi pathway. The discovery of hundreds of miRNAs has raised the overall field of biomedical RNAi to a striking level of the current recognition. Attention has been focused on the study of antisense oligonucleotide approaches to inhibit miRNA function and small interfering RNA-like technologies for the replacement of miRNAs. Scientists try to unravel the mystery of miRNA biology and explore its potential as therapeutic agents. Large high-throughput studies in patients have revealed that oncomiRs profiling can classify cancers and predict patient outcomes with high accuracy. High-throughput target analysis combining genomics, miRomics and proteomics might help delineating the spectrum of targets that are regulated by miRNAs. New knowledge about the functional roles of oncomiRs is revolutionizing cancer biology and will open up new opportunities in biomedical research.
